# Substitution of fishmeal: Highlights of potential plant protein sources for aquaculture sustainability

**DOI:** 10.1016/j.heliyon.2024.e26573

**Published:** 2024-02-20

**Authors:** Syed Makhdoom Hussain, Aumme Adeeba Bano, Shafaqat Ali, Muhammad Rizwan, Muhammad Adrees, Ameer Fawad Zahoor, Pallab K. Sarker, Majid Hussain, Muhammad Zubair-ul-Hassan Arsalan, Jean Wan Hong Yong, Adan Naeem

**Affiliations:** a Fish Nutrition Lab, Department of Zoology, Government College University Faisalabad, Punjab, 38000, Pakistan; b Department of Environmental Sciences, Government College University Faisalabad, Punjab, 38000, Pakistan; c Department of Biological Sciences and Technology, China Medical University, Taichung, 40402, Taiwan; d Department of Chemistry, Government College University, Faisalabad, Punjab, 38000, Pakistan; e Environmental Studies Department, University of California Santa Cruz, Santa Cruz, CA, 95060, USA; f Department of Fisheries and Aquaculture, University of Okara, Okara, Punjab, 56300, Pakistan; g Department of Life Sciences, Khawaja Fareed University of Engineering and Information Technology, Rahim Yar Khan, Pakistan; h Department of Biosystems and Technology, Swedish University of Agricultural Sciences, 23456, Alnarp, Sweden

**Keywords:** Fishmeal substitution, Plant products, Anti-nutritional factors, Physiological impacts, Sustainability

## Abstract

High protein content, excellent amino acid profile, absence of anti-nutritional factors (ANFs), high digestibility and good palatability of fishmeal (FM), make it a major source of protein in aquaculture. Naturally derived FM is at risk due to an increase in its demand, unsustainable practices, and price. Thus, there is an urgent need to find affordable and suitable protein sources to replace FM. Plant protein sources are suitable due to their widespread availability and low cost. However, they contained certain ANFs, deficiency of some amino acids, low nutrient bioavailability and poor digestibility due to presence of starch and fiber. These unfavourable characteristics make them less suitable for feed as compared to FM. Thus, these potential challenges and limitations associated with various plant proteins have to be overcome by using different methods, i.e. enzymatic pretreatments, solvent extraction, heat treatments and fermentation, that are discussed briefly in this review. This review assessed the impacts of plant products on growth performance, body composition, flesh quality, changes in metabolic activities and immune response of fishes. To minimize the negative effects and to enhance nutritional value of plant products, beneficial functional additives such as citric acid, phytase and probiotics could be incorporated into the plant-based FM. Interestingly, these additives improve growth of fishes by increasing digestibility and nutrient utilization of plant based feeds. Overall, this review demonstrated that the substitution of fishmeal by plant protein sources is a plausible, technically-viable and practical option for sustainable aquaculture feed production.

## Graphical abstract

**Figure:**
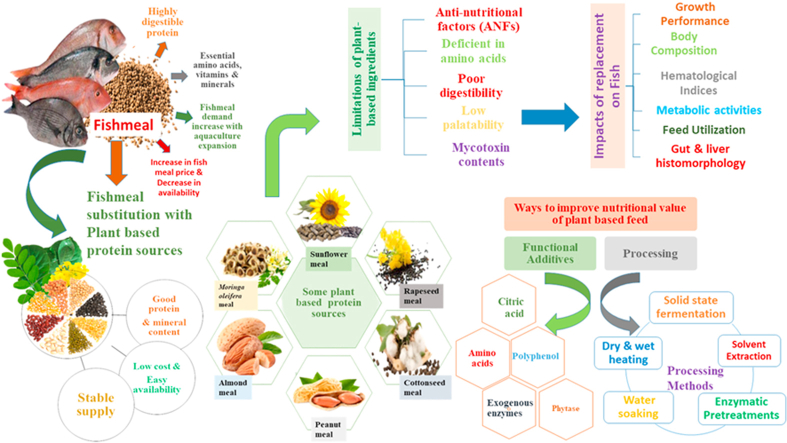

## 1 Introduction

Fish is a primary affordable protein source for over 950 million humans, providing about 16% of animal protein [1]. The high-quality and readily digestible fish protein with its unique content of eight essential amino acids (EAAs) is considered superior to other protein sources such as beef, milk, and eggs. Moreover, it is the principal source of omega-3 fatty acids, many vitamins, i.e. vitamin A, and minerals that are not available in many terrestrial protein sources. Fish and fish items form an essential part of the global food supply, meeting nutritional demands in both developed and developing countries [2].

With the rapid increase in world population and their demand for seafood as well, aquaculture is the best way to produce animal protein. Aquaculture can solve the problem of food shortage. Basically, it is the production of aquatic animals (fishes, crustaceans and mollusks) as well as aquatic plants under controlled conditions [3]. Aquaculture plays a crucial role in improving food security and generating home for fish farmers, particularly in developing regions. Furthermore, this industry is producing aquatic food at 7.5% growth rate per year since 1970 [4].

Fishmeal (FM) is used to feed aquatic animals. Nutrient rich FM is the best source of high quality protein with balanced amino acid content, omega-3 polyunsaturated fatty acids, minerals, vitamins (biotin, choline, vitamin A, D, E, and B12) as well as trace elements (iodine and selenium). It is highly digestible and palatable feed for farm animals. Commercial FM is mostly prepared from small, wild-caught sea fishes having high oil and bone percentage that are not appropriate for human use. A small proportion is also prepared from the byproducts of many seafood items made for human consumption. Major fishes used in a FM are shads, smelts, anchovies, sardines, menhaden, and herrings. High-quality FM usually has crude protein 60–72% and lipids are only 6–10% or 4–20% by weight as most oil is extracted during FM processing [5].

Aquafeed is a critical factor influencing the production of the aquaculture industry that affects its production. Exponential development of aquaculture to provide cheap protein to world's ever growing population has resulted an increase in demand for FM. Extensive overfishing for FM production led to a decline in marine species that further limits FM production [6]. Moreover, FM production has decreased due to restrictions on fishing. Due to all these factors, FM price has increased about 300% in recent years [7].

Expensive feed accounts for more than 50% of aquaculture production [8]. Overuse of FM also pollutes water owing to its high phosphorus content [9]. As FM is a limited world resource so its use as a single protein source for fish production is not viable. Therefore, now it is imperative to look for alternative protein sources to substitute FM for aquaculture sustainability. The current review highlights the significance of the use of different plant protein sources to replace FM. This review also focuses on the effects of using these products on different fish species. Moreover, the strategies to minimize the negative effects of plant based sources have also been discussed.

## 2 Fishmeal substitution with plant products

Plant protein sources are the best to substitute FM due to their ideal protein content, low cost, and stable supply from a large range of resources and are suitable feed for aquatic animals. Amino acid profile, energy density, fiber, and nutrient absorption are major factors for the substitution with plant based protein sources in aquafeed. However, plant proteins have some disadvantages over the FM that are a high proportion of cellulose [10], imbalance amino acid profile, and absence of some EAAs i.e. lysine, tryptophan, and methionine, and low palatability. Plants also have anti-nutritional factors (ANFs) i.e. phytic acid and inhibitors [11].

The use of alternative plant products will reduce feed cost and dependence on FM improving economic benefits. It is estimated that the aquaculture sector consumed 3.72 million tonnes of fishmeal (60.8 percent of global fishmeal production) in 2008. However, it is estimated that the aquaculture feed sector consumed about 6.8 million tonnes of soybean meal (25.1 percent of total compound aquafeeds by weight) in 2008. Other plant proteins being increasingly used include corn products, pulses, oilseed meals and protein from other cereals products. The total use of FM by the aquaculture sector is expected to decrease due to plant products [12]. These steps are pivotal for the sustainability of aquaculture [13]. This review explains some of the effective plant products such as soybean meal, black cumin seed meal, canola meal, lupin meal, rapeseed meal, rice, guar meal, almond meal and *Moringa oleifera* meal that has been utilized in fish feed. Conventional and non-conventional plant protein sources have been chosen for the novelty and uniqueness in research. Moreover, this review demonstrates the effects of substitution of plant ingredients with FM on different fish species.

## 3 Candidate plant products used in fish feed

### 3.1 Soybean meal

**Fig. 1:**
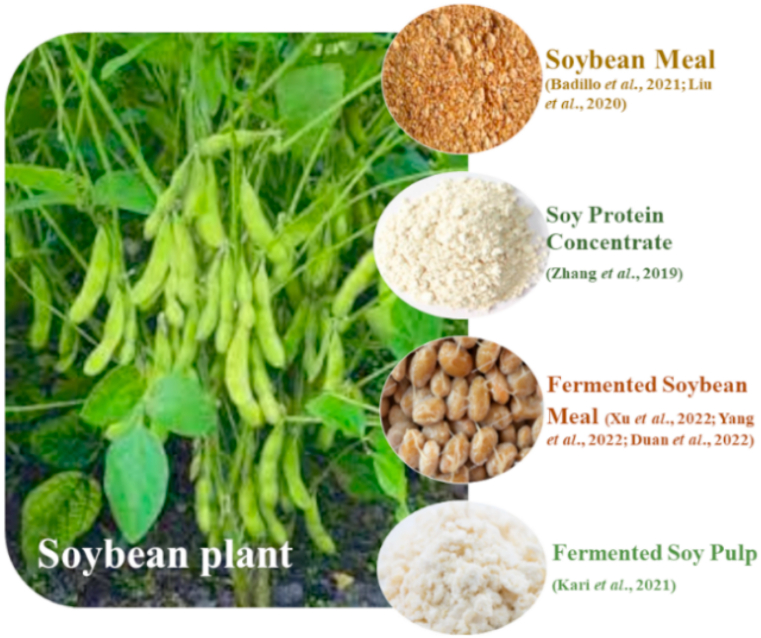
Different forms of soybeans used as fishmeal substitute.

Soybean meal (SBM) is first largest protein source having balanced amino acid profile with a low price and consistent supply making it an ideal to replace FM [14] (Fig. 1). Soybeans have about 41% protein and 20% ether extract based on dry matter [15]. However, SBM has low palatability, poor EAA content, and several ANFs i.e. phytates, tannins, trypsin inhibitors, and oligosaccharides [16]. Many studies have shown that SBM inclusion in high proportion in aquafeed affects the growth process, digestion, absorption, anti-oxidative capacity as well as causes damage to the gut of aquatic animals [17]. These negative effects can be minimized by fermentation of SBM to enhance probiotics and increase palatability. It also activates some components improving fish intestinal microbiota, increasing digestion and nutrient absorption [18] (Table 1).

**Table 1:** Soybean forms as substitute for fishmeal in diet of different fish species.

Fish Species	Soybean Forms	Tested inclusion levels	Duration	Optimum inclusion Level	Effects	References
Pacific fat sleeper *(Dormitator latifron)*	Soybean meal (SBM)	0, 40, 70, & 100%	60 days	100%	No significant difference in WG, SGR, SR up to 100% ↑ADC, Crude protein & lipid up to 100 % ↓Feeding cost	[155]
African catfish (*Clarias gariepinus)*	Fermented soy pulp (FSP)	0, 25, 50, 75 & 100 %	70 days	50%	↑ WG, SGR, RBC, LYM, LAB, TB up to 50% ↑ALB, GLOB, TP ↑FCR = ↑ FSP	[156]
Crussian carp *(Carassius auratus gibelio♀) ×* Common carp *(Cyprinus carpio♂)*	SBM	0, 20, 40, 60, 80 & 100 %	60 days	<38.52–41.81%	↑WG, SGR up to optimum level ↓HF, HMV, Digestive enzyme activity = ↑SBM ↑Fusobacteria, Proteobacteria & Actinobacteria ↓Firmicutes and Bacteroidetes	[157]
Obscure puffer (*Takifugu obscurus)*	Dehulled & defatted SBM + lysine, methionine, & taurine	0, 15, 30, 45, 60 & 75%	56 days	40%	↓ RBC, hemoglobin, methemoglobin, whole body lipid content ∞ ↑ SBM ↑ ALT & AST activity ↓GSH-Px & CAT activity	[26]
Amberjack *(Seriola dumerili)*	SBM + inosine	0, 25, 50 & 75% with or without inosine at 0.6%	56 days	50–75%	Inosine improve SBM utilization, innate immune responses, gut morphology & stress resistance against low salinity exposure TSP, ACH_50_, LA, BA, PA ↑ with inosine	[25]
Rice field eel (*Monopterus albus)*	Soy protein concentrate (SPC)	0, 15, 30, 45, 60 & 75%	56 days	26%	WG & FI ↓, FCR↑ over optimum level Improvement in T-AOC, SLP, digestive enzyme activity & alteration in growth-related genes expression pattern in skeletal muscle	[158]
Crucian Carp (*C. auratus)*	Fermented soybean meal (FSM)	0, 20, 40, 60 & 80%	56 days	40%	Serum T-AOC, POD & IgM ↑ upto 40% Significant differences in the midgut & hindgut microbiota	[23]
Largemouth bass (*Micropterus salmoides)*	FSM	Control, 75, 150, 225 & 300 g/kg	56 days	150 g/kg	↓WG, T-AOC, SOD, lipid & protein retention over optimum level ↑ FCR Gut *Cetobacterium*↓ & *Mycoplasma*↑ over 150 g/kg ↑ resistance against bacterial infection	[24]
Hybrid Snakehead (*Channa argus × Ch. Maculate)*	FSM	0, 72, 144, 216 & 288 g/kg	60 days	50 g/kg of FM replaced by FSM	WG & SGR ↓, FCR↑ ADC_DM_, ADC_CP_, PER, TC, intestinal muscle thickness & villus height decrease	[159]
Japanese seabass (*Lateolabrax japonicas)*	Conventional SBM + functional additives (FAS)	Control, 30% SBM without and with 0.0665% FAS	105 days	–	Growth, immunity, T-AOC & disease resistance ↓ at 50% FM replacement FAS ↑ antioxidant capacity & regulate gut microbiota	[27]
Turbot *(Scophthalmus maximus)*	Extruded full-fat soybean (EFS)	0, 10, 20, & 30%	60 days	20%	Serum LDL & TC, ADC_CP_, ADC_DM_, & gut probiotics reduced over 20% Water ammonia & nitrogen discharge, TSS, & fine solid accumulation increased over 20%	[160]
South American Catfish *(Rhamdia quelen)*	FSM	0, 7, 14, 21 and 28%	56 days	21%	↓ *Vibrionaceae* intestinal pathogenic bacteria up to 20 % No significant impact on gut lactic & heterotrophic bacteria concentrations, gut morphology & enzymatic activities	[22]
Turbot (*S. maximus* L.)	SBM or *L. acidophilus*-fermented SBM (LASM)	Control, SBM, LASBM	56 days	45% SFM + 1 × 10^8^ CFU g^−1^ (*L. acidophilus*)	SBM = ↓growth, ↓ activities of intestinal digestive & immune-related enzymes LASM = ↓ SM-induced negative effects on growth performance, morphology & microbiota, immune-related & digestive enzymes	[13]
Rainbow trout (*Oncorhynchus mykiss)*	Enzymatically hydrolyzed SM (ESM) + amino acid methionine & lysine (Met + Lys) + bile acid	Control, 45% ESM, E + A = ESM+0.76% (Met + Lys), E + B = ESM 0.02% bile acid, & E + AB = ESM + 0.76% (Met + Lys)+ 0.02% bile acid	56 days	–	ESM = ↓growth performance, ↑ liver health problem, ↓ lipid & SFA & ↑ protein load in muscle E + A = ↑growth performance, pepsin activity & fillet protein content E + B = ↓ growth & protease activity, ↑ amylase activity, ↑ SFA, MUFA & PUFA, ↑ lipid utilization E + AB = ↑ liver health	[161]

Abbreviations: ACH_50:_ Alternative complement pathways, ADC: Apparent digestibility coefficient, ADC_CP:_ Apparent digestibility coefficient of crude protein, ADC_DM:_ Apparent digestibility coefficient of dry matter, ALB: Albumin, ALT: Alanine aminotransferase, AST: Aspartate aminotransferase, BA: Bactericidal activity, CAT: Catalase, FCR: Food conversion ratio, GLOB: Globulin, GSH-Px: Glutathione, HF: Height of fold (hindgut), HMV: Height of microvillus (hindgut), IgM: Immunoglobulin M,LA: Lysozyme activity, LAB: Lactic bacteria, LDL: Low density lipoprotein, LYM: Lymphocytosis, MUFA: Monounsaturated fatty acids, PA: Peroxidase activity, PER: Protein efficiency ratio, POD: Peroxidase, PUFA: Polyunsaturated fatty acids, RBC: Red Blood Cell, SFA: Saturated fatty acids, SGR: Specific growth rate, SLP: Serum lipid profile, SOD: Superoxide dismutase, T-AOC: Total antioxidant capacity, TB: Total bacteria, TC: Total cholesterol, TP: Total protein, TSP: Total serum protein, TSS: Total suspended solid, WG: Weight gain.

Fermented soybean meal (FSM) is produced by introducing certain microorganisms into SBM for a fixed time period for fermentation by which ANFs are broken down and specific bioactive substances such as short peptides, probiotics, flavonoids, and organic acids are produced that improve the growth and health of aquatic animals [19]. Mostly, the solid state fermentation process is utilized to produce FSM that includes the grinding, steam sterilizing and adding of microorganisms for fermentation for a fixed period. Fungi and bacteria that are mostly used are *Lactobacillus, Aspergillus, Bacillus, Saccharomyces*, etc. Fermentation process, strains composition, and conditions show great impact on the FSM quality [20,21]. According to de [[22], [23], [24]], FSM significantly (*p<*0.05) improved the growth, intestinal morphology and increased disease resistance of south American catfish, crucian carp and largemouth bass, respectively.

Another way to improve effects of SBM is inclusion of functional additives such as lysine, methionine, taurine and inosine either separately or in combination [25,26]. A blend of antioxidants including ethoxyquin, a 1:1 standardized amalgam of carvacrol and thymol along with chelated trace elements, i.e., Mintrex® Cu, Zn and Mn, with conventional SBM when fed to Japanese seabass significantly improved the health and disease resistance as well as mitigate the adverse effects of SBM [27].

### 3.2 Cottonseed meal

**Fig. 2:**
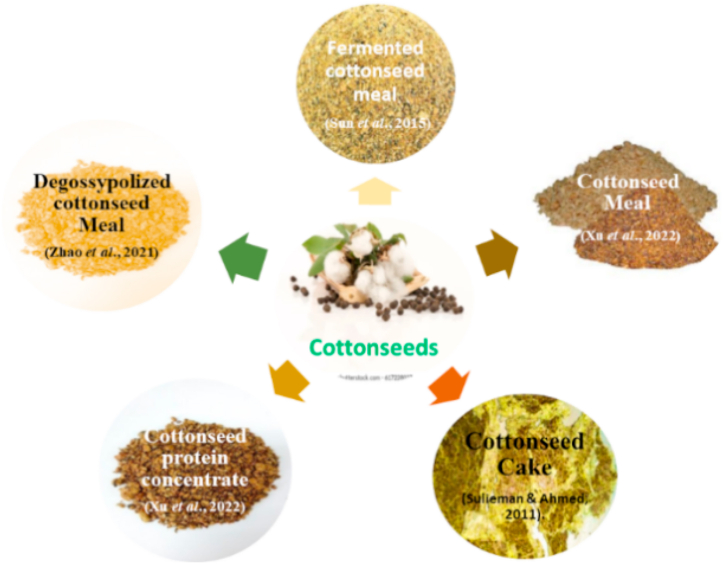
Different forms of cottonseeds that are suitable to replace fishmeal.

Cottonseed meal (CSM) is another plant protein source for fish feed due to its good protein content, high yield and low price (Fig. 2). Cottonseeds have about 51.20% crude protein, 9.3% ash, 1.6% ether extract, 7.02% fiber and 2.7 kcal/kg mass energy. Chemically active cyclopropenoid fatty acids, sterculic acid, and malvic acid are also found. But it is lysine deficient [28]. In 2013, the estimated cottonseed yield was 45.5 million tons in the world [2]. ANFs such as gossypol are found in cottonseeds that bound with lysine making its availability low to fish. Gossypol has negative impacts on the aquatic animals affecting their growth, fertility, and reproduction. Therefore, cottonseeds are converted to cottonseed protein concentrate through a large variety of processing methods i.e. dehulling, delinting, solvent extraction, and oil extraction at low temperature. These processes remove nonstarch polysaccharides, aflatoxins, and ANFs i.e. gossypol and protein denaturation is also greatly decreased [29,30] (Table 2). The study of [31] exhibited that cotton seed protein concentrate can replace 45% of FM without compromising health, growth and antioxidant status of largemouth bass. Similarly [32], revealed 10–50% replacement of FM in rainbow trout improved the growth, hematology, antioxidant parameters, and body composition by using concentrated dephenolization cottonseed protein.

**Table 2:** Cottonseed forms as substitute for fishmeal in diet of different fish species.

Fish Species	Cottonseed Forms	Tested Inclusion levels	Duration	Optimum inclusion Level	Effects	References
Largemouth bass (*M. salmoides*)	Cottonseed protein concentrate (CPC)	0, 15, 30, 45 & 70%	84 days	45% or 385.0 g/kg	WG↓, FCR↑, flesh composition & texture↓ over 45% ↓Serum glucose, TGC, TP at 70% Lower serum cholesterol content in all CPC levels	[31]
Rainbow trout (*O. mykiss*)	Concentrated dephenolization cottonseed protein	0, 10, 20, 30, 40 & 50 %	56 days	10–50%	At 10–50%, no negative impacts on growth performance, body composition, gut histomorphology, antioxidant & hematological indices ↑AOC at 30–50%	[32]
Silver sillago (*Sillago sihama Forsskál*)	Low-gossypol cottonseed meal (LCSM) + AA (methionine & lysine)	0,16, 32, 48 & 64 %	56 days	16%	WG& SGR ↑ up to 16% ↑expression of intestinal t TNF-α, NF-κB & IL-1β but ↓ZO-1, TGF-β3, IL-10 expression LCSM↑ = intestinal amylase activity↑ but intestinal trypsin activity ↓ & morphological damage to mid gut occur	[30]
Nile tilapia (*Oreochromis niloticus*)	CSM + exogenous protease	FM: CSM = 2:1, 1:1, 1:2, All three diets without or with 2500 U protease per kg diet	84 days	First & second group with protease	Protease improve growth performance, nutrient assimilation, & hematological indices Change in gene expression of GH & IGF-I Improve physiological indices	[162]
Southern flounder (*Paralichthys lethostigma*)	Genetically improved (GI) (glandless) & Genetically modified low-gossypol (GMO) CSM	0%, 50, 75 & 100% FM protein replace by GI or GMO-CSM protein, 100% R-CSM	56 days	75% GI or GMO-CSM	No effect on entire body omega-3 PUFAs, or liver gossypol up to 75% No adverse effect on body composition & growth performance up to 75%	[163]
Rohu (*Labeo rohita*)	Acidified phytase + CSM	0, 25, 50 & 75% with citric acid 0 & 2.5% & phytase 0 & 750 FTU/kg	84 days	50% with CA; 2.5%, PHY; 750 FTU/kg	Highest WG, SGR, FCR, crude protein & fat at optimum level Improvement in growth & body composition	[164]
Golden Pompano (*Trachinotus ovatus)*	CSM	0,20,40 & 60%	42 days	20%	Up to 20% no impact on muscle proximate composition & free EAA concentration Over 20% impaired glucose metabolism & muscle nutritive metabolism disorders	[165]
Turbot *(S. maximus* L.*)*	Low-gossypol CSM	0, 15, 25, 35 & 45%	56 days	35%	At 45% FER↓, PER↓, plasma glucose & cholesterol ↓, intestinal villi height ↓ ADCs of dry matter, crude protein & lysine ↓	[166]
Snubnose pompano *(T. ovatus)*	CSM + lysine & methionine	0, 25, 50, 75 & 100 %	70 days	100%	No negative impact on growth, metabolism, and general health at total replacement	[33]
Largemouth bass *(M. salmoides)*	CPC + yeast based paraprobiotic	Control, 23.5% CPC & 23.5% CPC +800 mg/kg yeast-based paraprobiotic	65 days	40% with yeast-based paraprobiotic	Improved growth performance Regulation in intestinal barrier & peptide transport related functions MsYF improve intestinal health by altering intestinal permeability, microbiota & inflammatory environment	[34]
Pearl gentian groupers (*Epinephelus fuscoguttatus♀ × E. lanceolatus*♂)	CPC	0 (FM, control), 10, 30 & 50 %	56 days	50%	No significant impact on WG, SGR, FCR, SR At 50% body lipid content ↑ Changes in genes related to hepatic lipid metabolism & enzyme activity	[167]
Black sea bream (*Acanthopagrus schlegelii)*	Fermented cottonseed meal (FCM)	0, 80, 160 & 240 g kg−1	56 days	16%	↓ SGR, WG, PPV, PER, & hepatosomatic index ↑ FCR, ADC_DM_ ↓ADCs of dietary protein and lipid	[168]

Abbreviations: AA: Amino acids, ADC: Apparent digestibility coefficient, AOC: Antioxidant capacity, EAA: Essential amino acid, FA: Fatty acid, FCR: Feed conversion ratio, FER: Feed efficiency ratio, GH: Growth hormone, IGF-I: Insulin like growth factor I, IL-10: Interleukin 10, IL-1β: Interleukin one beta, MsYF: Multi-strain yeast fractions, NF-κB: Nuclear factor kappa-light-chain-enhancer of activated B cells, PER: Protein efficiency ratio, PPV: Protein productive value, PUFAs: Polyunsaturated fatty acids, SGR: Specific growth rate, SR: Survival rate, TGC: Triglycerides, TGF-β3: Transforming growth factor beta-3, TNF-α: Tumor necrosis factor-α, TP: Total protein, WG: Weight gain, ZO-1: Tight junction proteins ZO-1.

Functional feed additives i.e. methionine, lysine and yeast based paraprobiotics are also largely supplemented with CSM to boost health and growth of fish [33,34]. Moreover, microbial fermentation of CSM also reduces concentrations of liver and dietary gossypol [35]. CSM fermentation with fungal mixture of *Saccharomyces cerevisiae* and *Candida tropicalis* for 48 h has improved its protein content from 20 to 33.5% [36] while fermentation with yeast has increased protein content from 8.745 to 12.67% [37].

### 3.3 Corn gluten meal

**Fig. 3:**
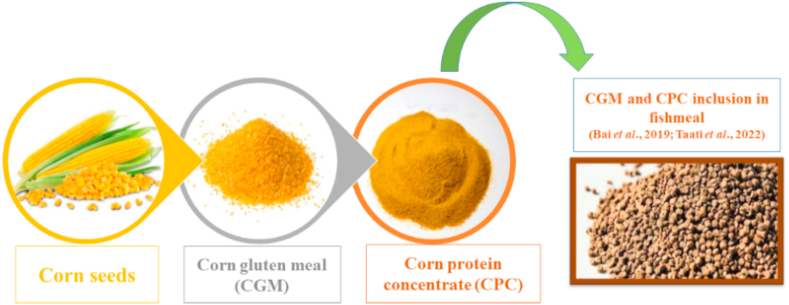
Different forms of corn seeds used to replace fishmeal.

Corn gluten meal (CGM) is obtained as a byproduct of wet grinding process for separation of protein, gums, fiber and starch parts from corn [38] (Fig. 3). It is beneficial plant protein source due to local availability, high protein content that is 60–70% of dry matter, low fiber content, and specifically due to low cost [39]. Moreover, it has few ANFs as compared to other plant based sources of protein [40]. In a study [40], demonstrated that 10% FM can be replaced by CGM in sea bass. The deficiency of EAAs i.e. lysine and arginine [41], and non-soluble carbohydrates i.e. raw starch up to 20% limit its inclusion in fish feed [42]. These non-soluble carbohydrates are reduced by enzymatic treatment and converted to corn protein concentrate (CPC) with high protein parts. Currently, there are two commercial types of CPC used in fish feed that are Lysto™ and Empyreal®75. The Empyreal®75 has higher methionine content (1.8 %) while lower in lysine (1.14 %) with crude protein 78%. Lysto™ has methionine 1.6%, lysine 6.2% with crude protein 77% [43] (Table 3).

**Table 3:** Corn products as substitute for fishmeal in diet of different fish species.

Fish Species	Corn Forms	Tested Inclusion Levels	Duration	Optimum inclusion level	Effects	Reference
Spotted rose snapper *(Lutjanus guttatus)*	CGM + crystalline Arginine & Lysine	0, 20, 40, 60, 80, or 100%	70 days	60%	↑Protein retention, ↓ lipid retention & ↓trypsin activity at 60 % Over 60% hypocholesterolemia & hypertriglyceridemia with negative effects on growth & FCR	[40]
Turbot *(S. maximus)*	CGM	0, 21.2, 31.8, & 42.6 % CGM to replace 0, 33, 50, & 67 FM protein	56 days	**-**	Negative impacts on growth & gut health by inducing enteritis ↓ intestinal immunity & AOC, ↓nutrient digestibility & feed utilization	[169]
Ussuri catfish *(Pseudobagrus ussuriensis)*	CGM	0, 10, 20, 30, 40, 50 & 60 %	56 days	40%	WG, SGR, FI, PER, ADCs ↓ over 40% but ADC of phosphorus ↑ ↑nitrogen excretion but ↓ phosphorus excretion Pepsin & serum lysozyme activity ↓ but ALT & AST activity ↑	[170]
Asian seabass *(Lates calcarifer)*	CGM	0, 5, 10, 15, & 20%	45 days	10%	Best FCR & ADCs up to 10% Crude lipid & gross energy highest at 20%	[171]
Siberian sturgeon (Acipenser baerii)	Corn protein concentrate (CPC)	0, 15, 30, & 45 %	56 days	15%	TL, FW, SGR, PER increase up to 15% FCR↓, SR = 100%	[172]
Rainbow trout *(O. mykiss)*	CPC	0, 30, 60, 90 & 120 g/kg CPC	56 days	81.0–82.2 g/kg	↑ Redness & yellowness of fillet ↑ ALT & LDH level at 120 g/kg ↑serum lysozyme activity ↓Proline & Threonine ∞↑ CPC	[173]

Abbreviations: ADC: Apparent digestibility coefficient, ALT: Alanine aminotransferase, AOC: Antioxidant capacity, AST: Aspartate aminotransferase, CPC: Corn protein concentrate, FCR: Feed conversion ratio, FW: Final weight, LDH: Lactate dehydrogenase, PER: Protein efficiency ratio, SGR: Specific growth rate, SR: Survival rate, TL: Total length, WG: Weight gain.

### 3.4 Rapeseed meal

**Fig. 4:**
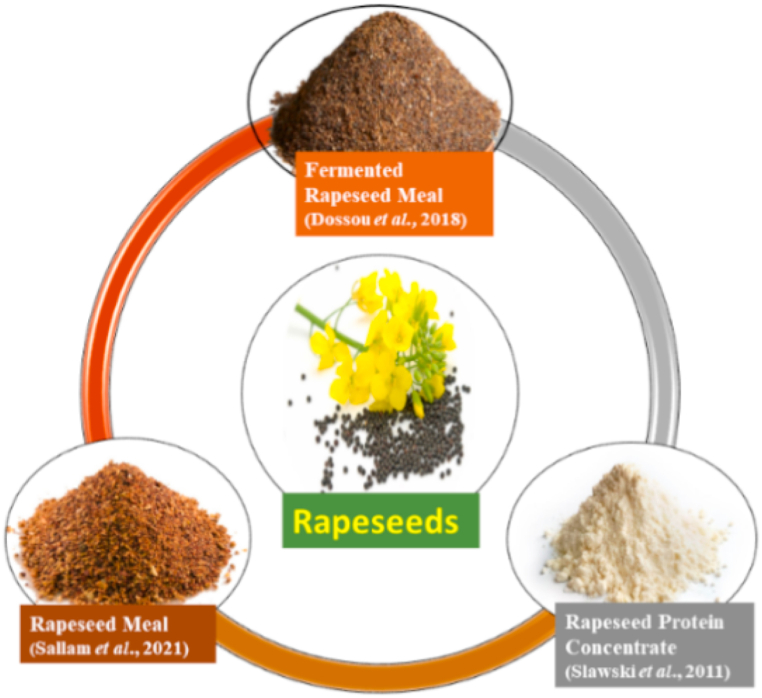
Different forms of rapeseeds used to replace fishmeal.

Rapeseed meal (RSM) has good protein content from 32 to 45% of dry matter, comparatively balanced amino acid content, low cost, and constant supply (Fig. 4). It also provides vitamins, minerals, and other microelements. It has lower lysine value than SBM but rich in sulfur amino acids [44]. Besides, RSM has a large number of ANFs i.e. tannis, phytic acid, sinnapine, erucic acid, glucosinolates, and indigestible carbohydrates. These factors show adverse effects on fish growth and health status [45,46] due to low palatability [47], poor digestibility and decrease in feed utilization [48]. In recent years, RSM yield has increased greatly becoming the second most important and commonly used protein source after SBM [49]. During microbial and enzymatic denaturation of rapeseed protein, certain functional bioactive peptides are released that perform specific biological activities including antihypertensive, immunomodulatory and antimicrobial [50,51]. Moreover, some degradation products from rapeseeds i.e. sinigrin and phenolic components, are observed having main antioxidant role with positive impacts in foods [52,53] (Table 4). Rapeseed protein concentrate (RPC) is also used in fish feed that is obtained after washing of RSM with aqueous ethanol [54]. For example [55], found that RSM, when fed to Nile tilapia and mango tilapia, showed improved weight gain and specific growth rate at 10% inclusion level while RPC replaces 33% of FM in Common carp.

**Table 4:** Rapeseed meal as substitute for fishmeal in diet of different fish species.

Fish Species	Rapeseed forms	Tested Inclusion levels	Duration	Optimum inclusion Level	Effects	Reference
Major carp *(Catla catla)*	RSM with Probiotic	34% RSM with 0, 1, 2, 3, 4 & 5 g/kg of probiotics	70 days	34% RSM with 2 g/kg of probiotics	High carcass composition, RBCs, WBCs, Hb & PLT at optimum level while low above optimum level Good immunological indices with 0, 1, 3 & 5 g/kg of probiotics	[174]
Nile tilapia (*O. niloticus) &* Mango tilapia *(Sarotherodon galilaeus)*	RSM	0, 10, 20, & 30%	84 days	10%	↑ FW, WG, SGR, WGR up to 10% ↑Mucosal & intestinal villi length and goblet cell number at 30% ↑AST & ALT with ↑RSM	[55]
Red Sea Bream *(Pagrus major)*	*Aspergillus oryzae* fermented RSM (RM-Koji)	0, 25, 50, 75 & 100%	56 days	50%	↑growth, nutrient assimilation & boosted immune responses & anti-oxidative impacts up to 50%	[175]
Asian red-tailed catfish *(Hemibagrus wyckioides)*	RSM	0, 11.2, 22.4, 33.6, & 44.8 %	56 days	11.2%	No negative impact on growth & health up to 11.2% ↓FGR, antioxidant capacity & digestive enzymes activities ↑Plasma aspartate aminotransferase & hepatic γ-glutamyl transferase activities	[103]
Ussuri catfish *(P. ussuriensis)*	RSM	0, 10, 20, 30, 40 & 50%	56 days	17%	↓FW, SGR, FI, FER, PER, ADCs = ↑RSM Hepatic AST, ALT & IGF-I gene expression level ↓ over 17% but muscles IGF-I gene expression level ↑ at 50%	[176]
Common Carp *(C. carpio* L*).*	Rapeseed Protein Concentrate (RPC)	0%, 33%, 66%, or 100%	56 days	33%	↓FI, FER, SGR over 33%	[177]
Rainbow trout *(O. mykiss* Walbaum)	RPC	0, 66 & 100%	84 days	–	No significant effect on growth performance, FI, FER, intestinal morphology & blood parameters	[178]
Rainbow trout *(O. mykiss)*	Rapeseed protein isolate	0, 33, 66 & 100%	56 days	66%	No significant effect on FCR, health parameters & body composition except ash content↓ ↓FI& FGR at total replacement	[179]

Abbreviations: AKP: Alkaline phosphatase, ALT: Alanine aminotransferase, AST: Aspartate aminotransferase, FA: Fatty acid, FCR: Feed conversion ratio, FER: Feed efficiency ratio, FGR: Fish growth response, FI: Feed intake, FW: Final weight, Hb: Hemoglobin, IGF-I: Insulin-like growth factor I, PLT: Platelets, RBC: Red blood cell, RSH: Rapeseed meal high, RSL: Rapeseed meal low, SGR: Specific growth rate, TG: Triglycerides, TP: Total protein, WBC: White blood cell, WG: Weight gain, WGR: Weight gain rate.

### 3.5 Canola meal

**Fig. 5:**
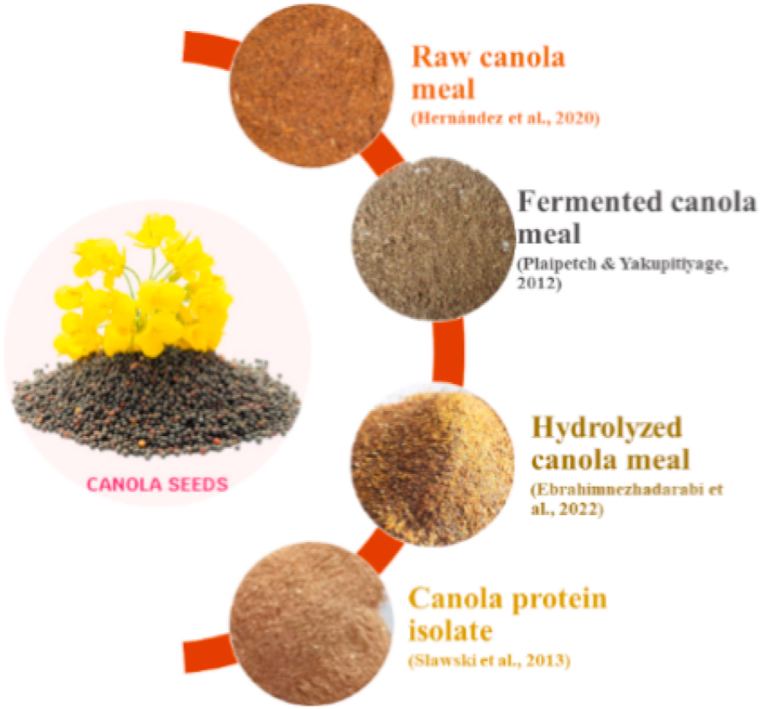
Different byproducts of canola seeds used as fishmeal replacers.

Canola meal (CM) is the solid matter left after the processing of canola seeds to extract oil [56] (Fig. 5). By weight up to 67%, CM is obtained from seeds after oil extraction [57]. It is most important source of plant protein as it has a minimum of 40% protein (dry matter) with easy availability and low cost. However, like other plant-based sources, it also contains ANFs i.e. non digestible oligosaccharides, glucosinolates and phytic acid, that limit its use in fish feed [48,58]. These factors have adverse impacts on fishes i.e. increase in gut viscosity and decrease in nutrient assimilation. High fiber content dilutes the density of nutrients [59], and phytates that are negatively charged make bonds with minerals decreasing their bioavailability [60]. The glucosinolates and their degraded products result in iodine deficiency, low feed intake, and increase in size of the thyroid gland, liver, and kidney [61].

Different techniques are used to lower or vanish ANFs that include alkaline and acid treatments, thermal extrusion [62], enzymatic process [63], microbial fermentation [64], and tail end dehulling [65]. All these methods are observed to decrease ANFs, but the benefits are restricted due to net protein loss, high processing cost, and/or partial reduction of ANFs. CM nutritional composition is greatly enhanced by fermentation using microbes to decrease ANFs [64] (Table 5) but due to high simple carbohydrate levels in CM, fermentation effect is limited to complex sugars. The removal of simple sugars by washing CM prior to fermentation increase microbial effect on complex carbohydrates i.e. fiber. These processes increase overall digestibility in fish species [66]. According to Refs. [67,68], CM when supplemented with phytase, citric acid and polyphenols, improved the nutrient digestibility, hematology and growth performance of *C. mrigala* and *C. carpio*, respectively.

**Table 5:** Canola meal as substitute for fishmeal in diet of different fish species.

Fish Species	Canola forms	Tested Inclusion levels	Duration	Optimum inclusion Level	Effects	Reference
Common carp *(C. carpio)*	Polyphenols + Canola meal (CM)	55% CM with 0, 100,200, 300, 400, 500 & 600 mg/kg	70 days	400 mg/kg of polyphenol with 55 % CM	Highest minerals absorption, best hematological parameters as well as proximate composition at optimum level	[180]
Mrigal carp *(Cirrhinus mrigala)*	CM + phytase & citric acid (CA)	0, 25, 50& 75 % without or with 750 FTU kg^−1^ & CA 2.5%	90 days	CM 50% with phytase 750 FTU kg-1 & CA 2.5%	Maximum WG & lowest FCR as well as best nutrient digestibility values at optimum level Dose dependent decrease in fish gut pH by CA	[67]
Nile tilapia *(O. niloticus)*	Processed canola meal (PCM)	0, 12.5, 25, 37.5 & 50%	36 days	25%	No effect on digestive enzymes, liver antioxidative status & mucosal immunity Liver SOD, GPx & CAT genes expression ↑ = PCM↑ Growth performance & intestine & liver tissue histoarchitecture ↓ over 25%	[181]
Blunt snout bream *(Megalobrama amblycephala)*	CM	208.2, 279.4, 350.5, 421.4, & 492.8 g/kg	112 days	50%	SGR, FER, PER ↑ but FI ↓ up to 50% PepT1 gene expression in gut ↑ then ↓ IGF-1 expression ↑ with ↑ CM Expression of TOR↓, AKT,4E-BP2 & S6K1 expressions ↑with CM ↑	[182]
Asian Sea Bass *(L. Calcarifer)*	Yeast fermented CM	25, 50, 75 & 100%	60 days	50%	↓ FW, PER, & nutrient digestibility while ↑ FCR over 50% ↓ Body crude protein, ash, Ca, Mg, P & their utilization with ↑ replacement	[183]
Beluga *(Huso huso)*	Hydrolyzed canola protein	0, 300, 400 & 500 mg of protein		500 mg	WG, SGR, PER & expression of genes highest up to 500 mg but lowest protein amount	[184]
Spotted rose snapper *(L. guttatus)*	CM	0, 150, 300, & 450 g/kg of CM protein	70 days	300 g/kg	↓SGR above optimum level ↑ CM protein = ↓FGR, ↓PER, ↓ ADC No effect on SR, FI & hematological parameters	[185]
Rainbow trout *(O. mykiss W.)*	Canola protein isolate	0, 25, 50%, 75 & 100%	70 days	75%	No negative impact on FGR, FI, FCR & palatability up to 100% ↑ WG, FCR, PER up to 75% No effect on SR & whole body composition	[186]

Abbreviations: 4E-BP2: Eukaryotic initiation factor 4E binding protein 2, ADC: Apparent digestibility coefficient, AKT: Protein kinase B, CAT: Catalase, FCR: Feed conversion ratio, FER: Feed efficiency ratio, FGR: Fish growth response, FI: Feed intake, FW: Final weight, GPx: Glutathione peroxidase, IGF-I: Insulin like growth factor I, PepT1: Peptide transporter 1, PER: Protein efficiency ratio, S6K1: Ribosomal protein S6 kinase 1, SGR: Specific growth rate, SOD: Superoxide dismutase, SR: Survival rate, TOR: Target of rapamycin, WG: Weight gain.

### 3.6 Peanut meal

**Fig. 6:**
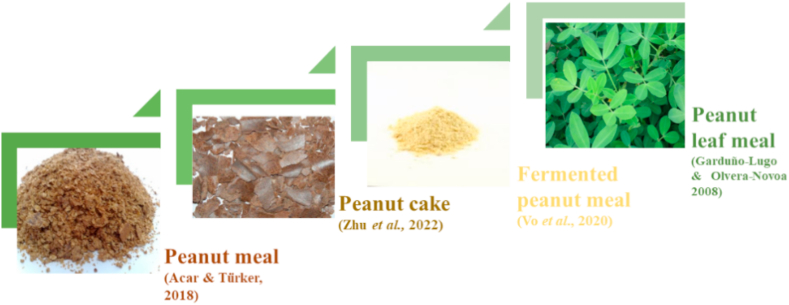
Different peanut products to replace fishmeal.

The peanut belonging to the legume family has high oil and protein content with high palatability as compared to other plant protein sources [69,70] (Fig. 6). The extracted peanut oil is used by humans [71] and left over peanut meal (PM) is rich in protein (55.94 %) and amino acid arginine (6.15%). It also has ANFs, i.e. trypsin, tannins, and amylase inhibitors, that show adverse impacts on fish health. Various methods are used to lower or eliminate these ANFs that are roasting and germination and a combination of both to enhance the peanut quality [72] (Table 6).

**Table 6:** Peanut products as substitute for fishmeal in diet of different fish species.

Fish Species	Peanut Products	Tested Inclusion levels	Duration	Optimum inclusion Level	Effects	Reference
Hybrid Grouper *(E. fuscoguttatus ♀* *× E. lanceolatus ♂)*	Peanut cake (PNK)	0, 6.6, 13.2, 19.8, 26.4, 33.0 & 39.6% of PNK by replacing 0, 11, 22, 33, 44, 55 & 66% of FM	56 days	15%	FCR & SGR ↑ over 33% TP first ↓ then ↑, TC ↓ Fat deposition in liver ↑ Harmful bacteria of gut ↑ over 44% WGR ↓ over 22%	[75]
Hybrid Grouper *(E. fuscoguttatus ♀ ×* *E. lanceolatus ♂)*	Peanut meal (PM)	0, 10, 20, 30, 40 & 50 %	70 days	50%	At higher levels ↑ in innate immune-related enzyme activity & gene expression, but ↓ antimicrobial peptide gene expression ↑ Gut pathogenic bacteria & ↓ Gut beneficial bacteria	[70]
Barramundi *(L. calcarifer)*	Germinated, fermented & untreated PM	15%, 30% & 60% GMP, FMP, UMP	56 days	60% FPM	At 60% GPM & UPM, ↑ liver lipid droplets & myodigeneration but ↓ acidic mucins in distal gut Stress increase plasma cortisol	[187]
Rainbow Trout *(O. mykiss)*	PM	0, 10, 20 & 30%	60 days	10%	No negative impacts on growth performance, feed utilization, hematological & serum biochemical parameters up to 10%	[188]
Nile tilapia *(O. niloticus* L.*)*	Peanut leaf meal	0, 10, 20 & 30%	126 days	20%	↓Growth performance & ↑ lipid content over 20% No effect on SR	[189]

Abbreviations: FCR: Feed conversion ratio, SGR: Specific growth rate, SR: Survival rate, TC: Total cholesterol, TP: Total protein, WGR: Weight gain rate.

Peanut cake, obtained from oil extraction process by squeezing oil [73], has great nutritional value specifically rich protein content in range of 410–450 g/kg (dry matter) but imbalance amino acid content. In comparison with PM, peanut cake contains slightly lower protein and higher lipid quantity, while other nutritional factors are same [74]. [70,75] investigated that 50% PM and 15% peanut cake, respectively, could be the optimum FM replacement levels when fed to hybrid grouper.

### 3.7 Guar meal

**Fig. 7:**
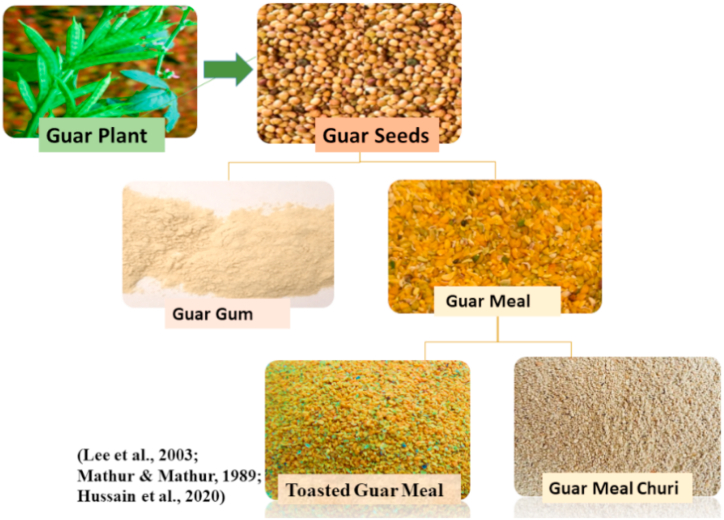
Different Guar products used for fishmeal replacement.

Guar, *Cyamopsis tetragonoloba,* from family Fabaceae, is a leguminous plant commonly called cluster bean (Fig. 7). It is low emission summer crop and used in feed of farm animals [76]. It occurs commonly in the East and South East of Pakistan and North and North West of India [77]. Guar meal is comparatively cheap high protein product gained after guar gum extraction that is valuable for producers of livestock [78]. Major ANF present is Galactomannan which is d-mannose polymer linked to d-galactose via α-1, 4 linkages attached to alternate β-1, 6 mannose units. This meal contains glactomannan 75–85%, moisture 8–14%, protein 5–6%, fiber 2–3% and ash 0.5–1%. The lowest crude protein present is 50% in guar meal in comparison with SBM which has 48% crude protein [79]. Guar meal contains tryptophan 0.68%, methionine 0.73%, cystine 0.79%, meth + cystine 1.51%, threonine 1.94%, isoleucine 2.31%, valine 2.35% lysine 3.22%, arginine 3.62%, and leucine 3.7% [80,81]. Guar meal is also free from aflatoxin, *Salmonella*, and *E. coli* as well as a good binding agent in feed formation [82]. It contains about 1.3 g per kg of saponin content [83]. Gum presence and ANFs limit its use in fish feed as an alternate plant protein source. Guar gum is largely utilized as thickner, stabilizer, and emulsifier in oil and food industries. After the gum extraction, guar seeds are toasted at high temperature to eliminate natural inhibitor trypsin that increases its digestibility and nutritional value [79] (Table 7). Similarly [84], found that when toasted guar meal was added to *L. rohita*, it substituted FM by 60%.

**Table 7:** Guar Meal as substitute for fishmeal in different fish species.

Fish species	Guar forms	Tested Inclusion levels	Duration	Optimum inclusion level	Effects	Reference
Rohu *(L. rohita)*	Toasted Guar Meal	0, 30, 60 & 90%	60 days	60%	Lowest crude protein & highest moisture, fat & gross energy at 90% while highest crude protein & lowest moisture, fat & energy at 30% Gut digestive enzyme activity ↓	[84]
Nile tilapia *(O. niloticus)*	Guar Sprout Meal	0, 25, 50, 75 & 100%	60 days	25–50%	↓GR, SGR, FCR, PER & feed utilization over 50% SR = 100%	[190]
Asian Catfish, (*Pangasianodon hypophthalmus)*	Guar meal	0, 5, 10, 15 & 20% guar meal to replace SBM at 0, 25, 50,75 & 100%	45 days	10%	FCR & SR ↓ over 10% Adverse impacts on growth performance & feed utilization over 10%	[191]
Rohu *(L. rohita)*	Enzyme treated guar meal	0, 25 & 50 % guar meal Pre-treated with Protease & multienzymes separately	60 days	25%	↑Growth with protease & multienzymes treated feed ↑ Gut digestive enzyme activities ↓carcass moisture & ash levels	[192]
Common Carp *(C. carpio* L.*)*	Guar meal	0, 25, 50, 75 & 100%	84 days	50%	↓ADC of protein, energy & lipid over 50% Significant differences in FW, SGR, FCR, FER, PER & FI	[193]
Mrigal carp *(C. mrigala)*	Guar meal with phytase enzyme & citric acid (CA)	Phytase 0, 500 & 1000 FTU kg-1 & 0, 2.5 & 5% CA with 56% guar meal in each dose	90 days	2.5% CA & 1000 FTU kg-1 with 56 % guar meal	Maximum growth performance, minerals & nutrient digestibility at optimum level ↓ Feed cost & nutrient's discharge via feces	[194]

Abbreviations: FCR: Feed conversion ratio, FER: Feed efficiency ratio FGR: Fish growth response, FI: Feed intake, FW: Final weight, PER: Protein efficiency ratio, SBM: Soybean meal, SGR: Specific growth rate, SR: Survival rate, growth rate, WG: Weight gain.

### 3.8 Sunflower meal

**Fig. 8:**
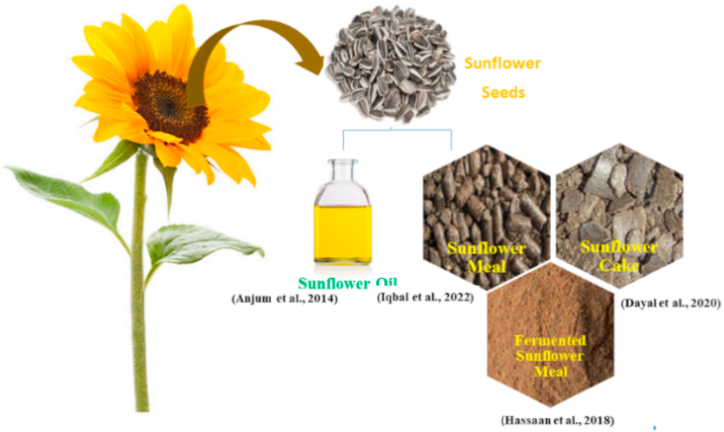
Sunflower seed byproducts as substitute of fishmeal.

Sunflower commonly called “Sooraj Mukhi” is fourth largest crop cultivated globally and contributes up to 30% of gross domestic production of cooking oil [85] (Fig. 8). Sunflower meal (SFM) is cheap protein and energy source. It comprises of oil extracted kernels that are rich in protein and sunflower hulls that have high fiber content about 180–230 g per kg [86]. It has 36–40% of crude protein with high tryptophan and methionine content [87]. In comparison with SBM, SFM contains lower lysine and sulfur amino acids but has other amino acids specifically aspartic and glutamic acid [88]. Main factors that restrict SFM use in fish feed are arginase and protease inhibitors [89], high fiber content and high phenolic compounds mainly caffeic and chlorogenic acids that also reduce solubility of protein [90]. ANFs can be inactivated by exogenous enzymes that improve digestion and absorption of nutrients of plant based protein for aquatic animals [91] (Table 8) [92,93]. found that 50% and 25% inclusion levels were optimum when SFM and fermented SFM were substituted, respectively.

**Table 8:** Sunflower Meal as substitute for fishmeal in diet of different fish species.

Fish species	Sunflower seed forms	Tested Inclusion levels	Duration	Optimum inclusion level	Effects	Reference
Nile tilapia *(O. niloticus)*	Sunflower meal (SFM)	0, 25, 50, 75 & 100%	90 days	50%	↑Growth performance & feed utilization but body indices ↓ up to 50% ↑whole-body protein, serum total protein &albumin up to 50% but whole body lipid, SGPT & SGOT activity increase over 50%, intestinal histology also effected by SFM	[92]
Nile tilapia *(O. niloticus)*	Fermented SFM with *Saccharomyces cerevisiae & Bacillus subtilis*	(0%), YFSFM (25, 50,75%) & BFSFM (25,50, 75%)	84 days	25%	Serum P & Ca, cholesterol, TGC & HDL lowest at 75% LDL & blood parameters not much effected ALT & AST values lowest at 25% Best growth performance & digestibility at 25%	[93]
Nile tilapia *(O. niloticus)*	SFM with exogenous xylanase	FM: SFM = (2:1, 1:1 & 1:2), each diet without or with 0.5 g/kg of exogenous xylanase	84 days	270g/kg with 0.5 g/kg of exogenous xylanase	Exogenous xylanase improved growth, digestive enzymes, nutrient digestibility, histological morphometric of liver & intestine and nutrient retention	[195]
Common carp *(C. carpio)*	SFM	0, 25, 50, 75, & 100%	70 days	75%	Highest WG at 25 % while lowest at 100% Significant decrease in TGC at 100% No adverse effects on growth, body composition, or hematological & plasma biochemical indices at 75%	[196]
Rohu *(L. rohita)*	SFM with Se Nanoparticles	50% SFM with 0, 0.5, 1, 1.5, 2, 2.5 & 3 mg/kg NPs	90 days	50% SFM with 1 mg/kg Se NPs	Best hematological parameters, nutrient digestibility, & growth performance at optimum level but highest SGR at 2 mg/kg NPs	[197]
Mrigal carp *(C. mrigala)*	SFM with Se Nanoparticles	50 % SFM with Se NPs0, 0.5, 1, 1.5, 2, 2.5 & 3 mg/kg	90 days	50 % SFM with Se NPs 1.5 mg/kg	Best body composition & increase in mineral absorption at optimum level Maximum Fe, Mn & Cr absorption at 2 mg/kg Highest absorption of Mg & Zn at 1 mg/kg	[198]

Abbreviations: ALT: Alanine aminotransferase, AST: Aspartate aminotransferase, Ca: Calcium, HDL: High-density lipoprotein, LDL: Low-density lipoprotein, P: Phosphorus, SGOT: Glutamic-oxalacetic transaminase, SGPT: Serum glutamic-pyruvic transaminase, SGR: Specific growth rate, TGC: Triglycerides, WG: Weight gain.

### *3.9* *Moringa oleifera* meal

**Fig. 9:**
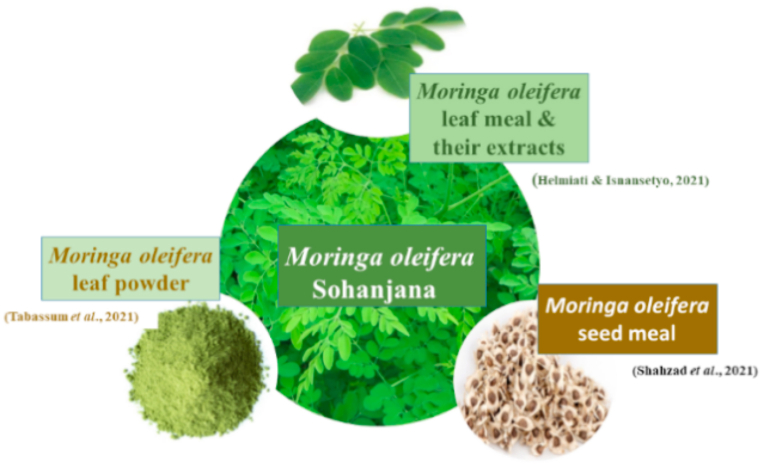
Different forms of *Moringa oleifera* used to substitute fishmeal.

*Moringa oleifera*, from family Moringaceae is a slender tree having softwood [94] (Fig. 9). It is largely found in Southern Punjab, commonly known as “drumstick or sohanjana” in Pakistan [95]. It is deciduous tree and ranges in size from 10 to 12 m [96]. In Pakistan, moringa leaves are utilized in tea, pods as vegetable, and roots to prepare pickle [97]. Typically, it is a multipurpose plant having several use in livestock, agriculture, pharmaceutics, human and other biological systems [98]. The pods and leaves have high mineral content like zinc, calcium, phosphorous, magnesium, manganese in small quantities, and are efficient source of vitamins (specifically C, B), proteins, amino acids, betacarotene, flavinoids and several phenolics [99]. Moringa fat free kernel and raw kernel meals contain 61.4% and 36.7% crude protein, respectively [60] while in leaves crude protein is 23–30% and crude fiber is less than 5.9% [100], resulting its good palatability for livestock and fish [101]. There are ten EAAs present in *M. oleifera* including lysine, histidine, leucine, isoleucine, valine, methionine, phenylalanine, tyrosine, tryptophan, and threonine [102]. ANFs present are tannins, saponins, lignins, phytates and oxalates. Tannins react with amylase and trypsin enzymes resulting non digestible compounds that decrease palatability and feed intake in fish [101]. These ANFs also give bitter taste resulting in low acceptability to aquatic animals [60]. The nutritional value of *M. oleifera* can be enhanced by fermentation process [103], phytase pretreatments [104], and by introducing organic acids i.e. citric acid to plant parts [68] (Table 9). For instance Refs. [68,105,106], exhibited that *M. oleifera* leaf meal (MOLM) replaced 10% FM, *M. oleifera* seed meal (MOSM) substituted 36% with 950 FTU per kg phytase and MOSM replaced 35% with 3% citric acid, respectively.

**Table 9:** *Moringa oleifera* as substitute for fishmeal in diet of different fish species.

Fish Species	*Moringa oleifera* forms	Tested Inclusion Doses	Duration	Best Inclusion Level	Effects	Reference
Mrigal carp *(C. mrigala)*	*Moringa oleifera* leaf meal (MOLM)	0, 10, 20, 30, 40 & 50%	90 days	10%	Maximum growth performance & nutrient digestibility at 10% RBCs, WBCs, Hb ↓ = ↑ MOLM	[105]
Common carp *(C. carpio)*	*Moringa oleifera* seed meal (MOSM) + MOLM with phytase pretreatment	36% moringa + phytase levels 0, 500, 650, 800, 950, 1100 & 1250 FTU kg^−1^	70 days	36% with 950 FTU per kg	↑ WG, SGR, FCR, nutrient digestibility & mineral absorption up to optimum level ↓ Discharge of minerals & nutrients in water bodies	[199]
Mrigal carp *(C. mrigala)*	Citric Acid Acidified MOSM	35 % MOSM with 0, 1, 2, 3, 4 & 5% CA levels	90 days	35% with 3% CA	Improved mineral absorption, carcass composition & hematological parameters up to optimum level, Maximum crude protein & fat content up to 3%	[68]
Nile tilapia *(O. niloticus)*	MOSM + MOLM with phytase	36% moringa with PHY levels 0, 500, 650, 800, 950, 1100 & 1250 FTU per kg	70 days	36% with 950 FTU per kg	Maximum values of RBCs, PLT & Hb at optimum level Enhanced hemato-immunological parameters, body composition, fish health & growth gene expressions	[106]
Gibel carp *(C. auratus gibelio)*	Fermented *Moringa oleifera* leaf meal (FMOLM)	a basal diet with 10% FM & others 20, 40, 60	50 days	40%	↑ growth, antioxidant & immune response & regulate expression of immune-related genes & ↑ disease resistance against *A. hydrophila* via TLR2 pathway up to 40%	[200]
Red tilapia (*Oreochromis* sp.*)*	FMOLM	0, 10, 20 & 30 %	60 days	20%	At 20%, hematocrit & leukocrit ↑, phagocytic activity & phagocytic index ↑ but suppression in monocyte Increase in lymphocytes & total plasma protein	[201]
Catfish *(C. gariepinus)*	MOLM	0, 0.5, 1.0 & 1.5%	56 days	1.5%	Best hematological parameters at 0 & 1.5% hematological performance increase by MOLM	[202]
Rainbow trout *(O. mykiss)*	MOLM	0, 10, 20, 30 & 40%	90 days	20%	↑ WG & SGR but ↓ FCR up to 20% ↑ Blood Proteins & SOD, CAT, & GPx activity ↓AST & ALT activity ↑ IL-6, IL-8 & IL-10 expression compared to β-actin gene	[203]

Abbreviations: ALT: Alanine aminotransferase, AST: Aspartate aminotransferase, CAT: Catalase, FCR: Feed conversion ratio, GPx: Glutathione peroxidase, Hb: Hemoglobin, IL-10: Interleukin −10, IL-6: Interleukin −6, IL-8: Interleukin −8, PLT: Platelets, RBC: Red blood cell, SGR: Specific growth rate, SOD: Superoxide dismutase, TLR2: Toll-like receptors 2, WBC: White blood cell, WG: Weight gain.

### 3.10 Almond meal

**Fig. 10:**
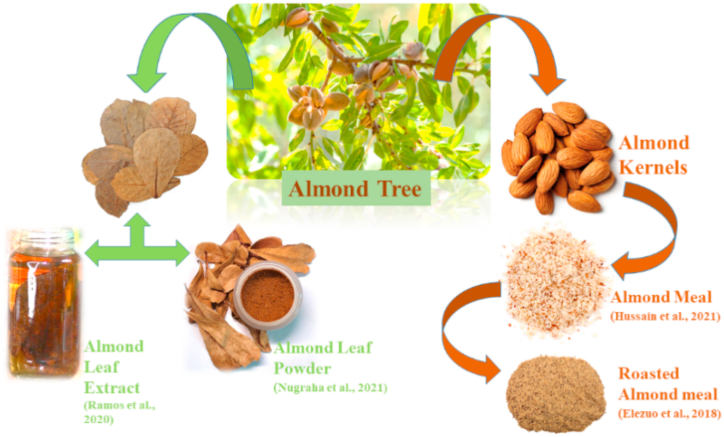
Different almond meals used to replace fishmeal.

*Terminalia catappa*, from family Combretacea, is commonly known as wild almond, sea almond, Indian almond or tropical almond (Fig. 10). The annual fruit production is about 700,000 tons worldwide [107]. It is mostly found in Taiwan, the Malay Peninsula, tropical Asia and India. Almond nut or seed, *Prunus amygdalus*, is categorized into two groups: (1) Bitter almond “*Prunus amygdalus amara*” (2) Sweet almond “*Prunus amygdalus dulcis*” [108]. The sweet almonds provide edible nuts [107]. Almond kernels have brown skin layer covering with an intermediate shell and an outer hull. They have 24.5% crude protein, 6% ash, 36% crude protein and rich in vitamin E as well as phenolic compounds. Almond meal also contains considerable oil quantity because extraction of oil is done by cold pressing method. Owing to unique antioxidant effects, almond meal boosts immune system, making it effective substitute for FM [109]. Available research data has shown that almond meal is under study as a possible alternative of FM in diet of different fish species (Table 10). Up to 40% almond meal based diet significantly improved the growth performance, nutrient absorption and hematology of *L. rohita* [68].

**Table 10:** Almond meal as substitute for fishmeal in diet of different fish species.

Fish species	Almond forms	Tested inclusion levels	Duration	Optimum inclusion Level	Effects	Reference
Nile tilapia *(O. niloticus)*	Almond leaf meal	0, 25, 50, 75 & 100%	70 days	25–100%	↑ WG, SGR, FCR ↑ survival against *Salmonella typhi*	[204]
Rohu *(L. rohita)*	Almond meal	0, 20, 40, 60, 80 & 100%	70 days	40%	Significant ↑ in FGR, nutrient digestibility & hematology up to 40% Hb & RBCs ↓ over 40%	[205]
Snakehead *(Ch. striata)*	Almond leaf powder	0, 0.25, 0.50 & 0.75 g L-1	40 days	0.50 g/L	↑ survival, SGR, FCR, PER Normal hematological & physiochemical parameters up to 0.5 g/L	[206]
Leaf fish *(Monocirrhus polyacanthu*	Almond leaf extract	0.25, 0.50, 0.75, 1.00 & 1.25 g/L	15 days	0.75 g/L	↓Mortality & ↑ growth performance ↑WG, SGR, length, survival up to 0.75 g/L ↑water quality parameters value	[114]
African catfish *(C. gariepinus)*	Roasted tropical almond meal	0, 25, 50, 75 & 100%	105 days	75%	↑WG, ↑SGR, ↓ FCR at 75% ↓WG, ↓SGR & ↑FCR at 100% Economic depression over 75%	[109]
Cardinal tetra (*Paracheirodon axelrodi)*	Tropical Almond leaves	0, 0.5, 1.5 & 2.5 g/L	**_**	0.5–1.5 g/L	↑WG, length, survival (100%)	[115]

Abbreviations: FCR: Feed conversion ratio, FGR: Fish growth response, Hb: Hemoglobin, PER: Protein efficiency ratio, RBCs: Red blood cells, SGR: Specific growth rate, WG: Weight gain.

*Terminalia catappa* leaf extract (TCE) has been utilized in culture of aquatic animals by farmers as an eco-friendly substitute due to high levels of tannins and phenolic compounds [110]. Although it controls fungal, parasitic and bacterial infections [[111], [112], [113]], it also works as a behavioral stimulator for spawning substrate and reproduction [114]. Improvement in growth of fish, survival rate as well as water quality parameters have been noticed by TCE use for Leaf fish, *Monocirrhus polyacanthus,* & Cardinal tetra, *Paracheirodon axelrodi,* [114,115].

### 3.11 Black cumin seed meal

**Fig. 11:**
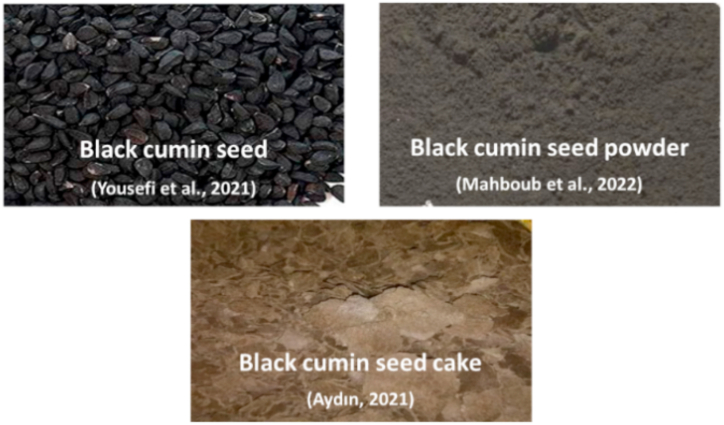
Different Black cumin seed forms used to replace fishmeal.

*Nigella sativa*, commonly called black cumin seed is from family Ranunculacea and is a medicinal herb with remedial characteristics (Fig. 11). It has been utilized for therapeutics purposes for over 2000 years. *N. sativa* is indigenous to Southern Europe, Southwest Asia and North Africa and is cultured in different regions of world including Middle Eastern Mediterranean region, Saudi Arabia, Turkey, Pakistan, India, and South Europe [116,117]. Therapeutic effects of black seeds have been recorded as anticancer, antihistaminic, antidiabetic, antioxidant, antiviral, antifungal, antiprotozoal, antibacterial, anticholesterol, anti-inflammatory and immonomodulator [118]. Black seed comprise of crude protein 20.8%, lipids 34.8%, ash 3.7%, carbohydrate 33.7% and 7.0% moisture [119]. Black seeds and oil have been utilized commonly for treating many health conditions belonging to immune, respiratory and cardiovascular system, digestive tract, liver and kidney functions [117]. Most of its pharmacological characters are because of thymoquinone (18.4–24.0%) that is main bioactive element of its volatile oil (0.4–2.5% of seed oil) [117,120]. Black cumin seed cake (BCSC) is a byproduct of agro-industry obtained after oil extraction from seeds and is nutritionally valuable for animals. It has crude protein 27.5% and crude oil 14.8% [120]. As a whole, owing to local availability, low price, and effectiveness, the use of *N. sativa* and its oil in aquaculture has been urged as a feed ingredient for better fish health, making them more resistant to bacterial infections such as *Burkholderia cepacia* [121] and *Aeromonas hydrophila* [122] (Table 11).

**Table 11:** Black cumin seeds as substitute for fishmeal in diet of different fish species.

Fish species	Black Seed forms	Tested Inclusion levels	Duration	Optimum inclusion Level	Effects	Reference
Nile tilapia (*O. niloticus)*	Black cumin powder against *Burkholderia cepacia*	30 g/kg of NS-incorporated diet	45 days	**_**	Protective effects on immune response, antioxidant capacity, hepato-renal function, modulation of gene expression, antibacterial activity against *Burkholderia cepacia*	[121]
Tilapia *(O. niloticus)*	Black cumin flour against *Aeromonas hydrophila*	0, 20, 35, 50 and 65 g/Kg	90 days	50 g/kg	Boosted the immune system	[122]
*Mirror carp (C. carpio* var. *specularis)*	Black cumin seed cake (BCSC)	SBM replaced by 0%, 25% & 50% of BCSC	63 days	< 25%	↑BCSC level = ↓Growth & feed intake No significant impact on serum total protein, globulin, ALT, AST & glucose ↓ Cholesterol & triglyceride at > 25% No significant impact on palmitic acid, stearic acid, linolenic acid, linoleic acid, SFA, MUFA & PUFA of muscle	[207]
Common carp *(C. carpio)*	Black Seed	Control, 0.25, 0.5 & 1%	60 days	**_**	Black seed remove negative effects of glyphosate exposure Stable levels of biochemical serum parameters & cholesterol, higher levels of immune defences & antioxidant enzymes, lower lipid peroxidation, metabolic enzymes & cortisol levels than control fish	[208]
Nile tilapia (*O. niloticus)*	Black seed meal (BSM)	0, 25, 50, 75 & 100% of BSM alternative to SBM	60 days	50%	↓Growth performance & feed utilization above 50% No impact on carcass crude protein ↑Ash content = ↑ BSM Feed costs decrease upto 50%	[209]
Rainbow trout *(O. mykiss)*	Methanolic extract of black cumin	0, 0.1 & 0.5 g/kg	30 days	_	Stimulate some innate humoral immune responses, but ineffective for cytokine-related gene trancriptions, ↓FCR, ↑Respiratory burst activity	[210]
Rainbow Trout *(O. mykiss*, Walbaum)	Black Cumin Seed Powder	0, 0.5, 1, 2.5, 5, 10 & 20 g/kg	60 days	_	Positive differences in plasma protein, MCH, MCHC, RDW_SD, PLT and MPV at all levels	[211]
African catfish *(C. gariepinus)*	Black seed	Control, 4-nonylphenol (NP)-treated, 1, 2.5 & 5% black seed treated groups	21 days	_	Black seed with 4-NP significantly decrease nephrotoxic effect of 4-NP & sustain normal kidney function & structure	[212]

Abbreviations: SBM: Soybean meal, ALT = Alanine aminotransferase, AST = aspartate aminotransferase, SFA=Saturated fatty acids, MUFA = Monounsaturated fatty acids, PUFA= Polyunsaturated fatty acids, FCR= Feed conversion ratio, MCH = Mean cell hemoglobin, MCHC = Mean cell hemoglobin concentration, RDW-SD= Red cell distribution width, PLT= Platelet, MPV = Mean platelet volume.

### 3.12 Lupin meal

**Fig. 12:**
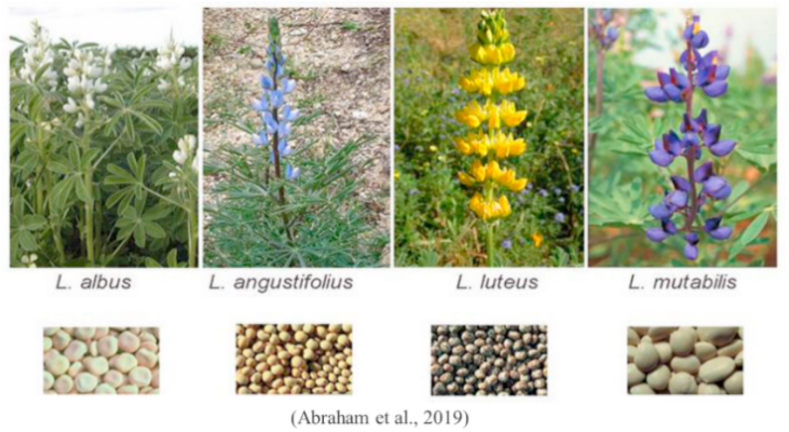
Mostly cultivated lupin species.

Lupins plants from genus lupinus are found worldwide having many species but mostly cultivated species are only four including narrow-leaf lupin (*L. angustifolius*), white lupin (*L. albus*), pearl lupin (*L. mutabilis*) and yellow lupin (*L. luteus*) [123] (Fig. 12). Lupin seed meals (LSMs) are included as supplement globally in livestock feed formulation due to its good nutritious value and potential to grow on infertile lands [124]. LSM has good protein content, high dietry fiber and locally available at low price [125]. Comparatively, they have low ANFs including tannis, phytic acid, oligosaccharides and alkaloids than present in other plant based ingredients. Owing to their availability and suitability, LSMs are routinely utilized in aquafeed [126]. Pretreatment of lupin with *Lactobacilli* enhanced its nutritional value as well as micronizatin process increased starch digestibility and destroyed the ANFs [127,128]. The advantageous influence of lupin in comparison with FM has been prescribed in diets of different fish species (Table 12). Lupin meal can be used in rainbow trout up to 30% without adverse effects on growth performance, hematological and serum biochemical markers [129].

**Table 12:** Lupin meal as substitute for fishmeal in diet of different fish species.

Fish Species	Lupin forms	Tested Inclusion levels	Duration	Optimum inclusion Level	Effects	Reference
Rainbow trout *(O. mykiss)*	Lupin meal	0, 15, 30, 45, & 60%	60 days	30%	Best growth performance upto 30 % ↓ Heamatocrit & MCV at 60% ↓TP, TGC, cholesterol, ALP, LDH in all treated groups	[129]
Cobia *(Rachycentron canadum)*	Lupin kernel meal (LKM)	0, 200, 400, 600 & 800 g/kg of FM replaced by LKM	56 days	200 g/kg of FM replaced by LKM	↓FBW, SGR, FI & ADCs = ↑LKM ↓Haematological indices & histopathological changes ↑ AST & ALT	[213]
Nile tilapia (*O. niloticus)*	Different UK lupin meal cultivars	Yellow lupin, blue lupin, fermented yellow lupin & fermented blue lupin	49 days	Dehulled (kernel meal) *Lupinus luteus* cv. Pootalong	Significant increase in WG, SGR, FCR, PER and K with fermented meals Improved enterocyte height	[214]
Barramundi *(L. calcarifer)*	Fermented lupin	0, 30, 45, 60 & 75%	61 days	_	Fermentation decresed anti-nutrients & upgraded amino acid profile of lupin ↑ in final weight & length of Barramundi	[128]
Gilthead sea bream (Sparus aurata L.)	Micronized lupin seed meal (MLSM)	10, 20 & 30% of raw lupin & 20 & 30% MLSM	84 days	MLSM	MLSM promote significantly higher growth rates than raw lupin seeds	[127]

MCV = Mean cellular volume, TP = total protein, TGC = triglyceride, ALP = alkaline phosphatase, LDH = lactate dehydrogenase, FBW = final body weight, SGR = specific growth rate, FI = feed intake, ADCs = Apparent digestibility coefficients, ALT = Alanine aminotransferase, AST = aspartate aminotransferase, WG = weight gain, SGR = specific growth rate, FCR = feed conversion ratio, PER = protein efficiency ratio K = condition factor.

### 3.13 Rice (rice protein concentrate and rice distiller's grains)

**Fig. 13:**
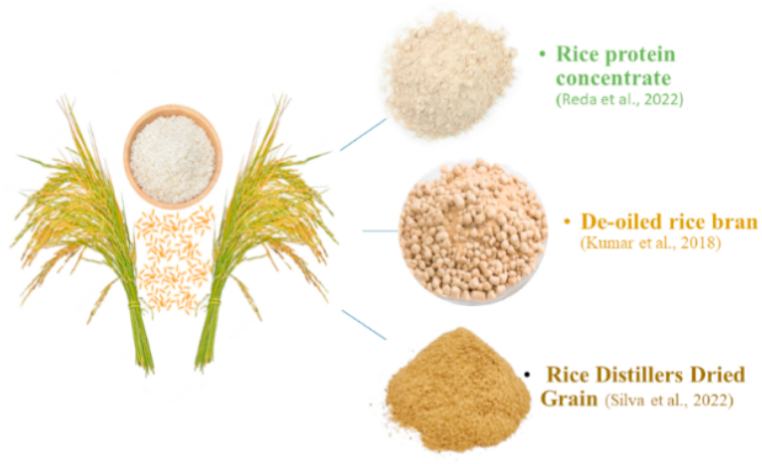
Rice byproducts used to replace fishmeal.

Rice is an important food crop globally. Rice protein concentrate (RPC), a byproduct of rice processing, has great nutritional value as it contains about 66–70% protein and 10–11% lipid, becoming an ideal replacer of FM (Fig. 13). But, it is deficient in lysine that limits its use in aquafeeds. Recently, RPC has become available at commercial level for use in fish and animal feed [130,131]. RPC can replace up to 25% of the FM protein in the diet of *O. niloticus*, enhancing the antioxidant capacity, immunocompetence, and disease resistance [132]. Enzymatic rice protein is a treated product of RPC via action of polysaccharides and proteases have many monosaccharides and short peptides which improve digestion and growth [133,134]. This processing also reduces adverse effects of rice protein due to disulfide bonds i.e. reduction in growth as well as immune response of fishes [135,136]. Fermented rice protein (FRP) is obtained by microbial fermentation has high acid, good solubility, low viscosity and heat stability [137]. Rice or rice bran fermentation by different types of bacteria is reported for use as food for animals. However, only few studies on use of FRP in aquatic animals feed are present. Another byproduct is rice distillers dried grain (rice DDG), obtained from rice grains during ethanol production, have 70.4% crude protein (about 3.6% lysine), 2.9% crude fiber and 9.7% lipid [138], also utilized in aquafeed. De-oiled rice bran (DORB) comprise of 6–10% of rice by weight with greater nutritional value than many other agri-products. It is free of fat, also called rice polish being good substitute for FM [139] (Table 13). DORB has crude lipid 0.33%, crude protein 15.30%, crude fiber 14.45%, nitrogen free extract 63.88% and ash 6.01% [140].

**Table 13:** Rice as substitute for fishmeal in diet of different fish species.

Fish species	Rice forms	Tested Inclusion levels	Duration	Optimum inclusion Level	Effects	Reference
Nile tilapia (*O. niloticus)*	Rice protein concentrate	0, 25, 50 & 75%	5 months	25%	↓Antioxidant capacity & immune indices over 25 % ↑mRNA levels of intestinal cytokines at 25% ↓ Gut microbial diversity ↑ Immunocompetence & disease resistance	[132]
Channel catfish *(Ictalurus punctatus)*	Enzymatic rice protein	0, 2.5, 5.0 & 7.5%	56 days	2.5 %	Improved intestinal morphology & digestion at 2.5% Upset gut microbiota equilibrium, results in damage to gut mucosa & inflammation at 7.5%	[215]
Hybrid groupers *(E. fuscoguttatus ♀× E. lanceolatus♂)*	Fermented rice protein (FRP)	10, 30 & 50 %	56 days	10%	↑Activities of intestinal digestive enzymes & brush border enzymes by FRP ↓Expression of immune-related genes & relative abundance of *Enterococcus & Bacteroides*	[216]
Silver catfish *(Rhamdia quelen)*	Rice ethanol distillery residue	0, 25 & 50%	60 days	_	Reduced final growth, deposition of body protein & increased free liver amino acids	[217]
Red Seabream *(P. major)*	Rice Distillers Dried Grain	0, 5, 10, 15, 20 & 25%	70 days	25%	No negative effects on overall body performance	[218]
Rohu *(L. rohita)*	De-oiled rice bran	0, 20, 30, 40, 50 & 60%	60 days	40%	Significant effect on digestive, metabolic enzyme activities & haematological parameters High oxidative stress activity at 60% increased metabolic activity & immune responses upto 40%	[219]

### 3.14 Brewer's spent grains and spent yeast

The breweries produce large volumes of byproducts including spent grains, spent yeast and spent hops, that are potentially used as aquafeed. The recent exponential rise in number of global small brewing industries produce huge amount of byproducts which are available at low or no price. Brewers' spent grains (BSG) are the biggest byproduct of beer brewing process accounts for 70–85% of total byproducts, almost 1/3 of total malt weight from production of beer, and 40 million MT of BSG are generated worldwide annually. BSG is a lignocellulosic residue that mainly comprise of fiber (30–50%), protein (19–30%) and also lipids, vitamins and minerals [[141], [142], [143], [144]]. Brewers’ spent yeast (BSY) is the second main byproduct from brewing process and also a source of protein (40%–50% DW) minerals and vitamins [145], and several other bioactive elements including mannan oligosaccharides, b-glucans, and nucleic acids [146]. Many studies have shown that BSG and BSY can successfully substitute a part of dietary fishmeal in different fish species (Table 14), such as in *Oncorhynchus mykiss* where, 15% BSG + BSY and 20% yeast can replace FM.

**Table 14:** Brewery byproducts as substitute for fishmeal in diet of different fish species.

Fish species	Brewery byproducts	Tested Inclusion levels	Duration	Optimum inclusion Level	Effects	Reference
Rainbow trout (*O. mykiss*)	Dry & hydrolyzed spent yeast (DSY & HSY) & spent grains (DSG & HSG)	10 & 20% of DSY, HSY & 7.5% & 15% of DSG & HSG to the basal mixture	60 days	20% yeast & 15% spent grain	↑ final weight, protein digestibility & food conversion, No change in muscle nutritional composition	[220]
European seabass *(Dicentrarchus labrax)*	Fermented brewer's spent grain (BSG)	Control, 0.4 & 0.8% BSG-extract pretreated + direct inclusion	66 days	Pretreated 0.4% BSG-extract	↑ Dietary reducing sugars content, antioxidant activity & cellulase and xylanase activities No effect on growth performance & whole-body composition ↑Feed efficiency & protein utilization	[221]
Rainbow trout (*O. mykiss*) & Gilthead seabream (*S. aurata*)	Spent yeast & Spent grain	Dry & hydrolyzed spent yeast & spent grains	30 days	20–30% brewers' spent yeast & spent grain	Good protein, lipid & amino acid digestibility	[222]
Striped catfish, *(Pangasianodon hypophthalmus)*	Brewer's spent grains (BSG)	SBM replaced by 0, 25, 50, 75 or 100% of BSG		50%	Improvement in growth performance, nutrient utilization and feed conversion upto 50%	[223]
Nile Tilapia, *(O. niloticus)*	Brewer's waste	25, 50, 75 & 100%	70 days	50%	↓ Feed intake & utilization with increase level no adverse effect on growth and feed utilization upto 50%	[224]
Thai Panga (*P. hypophthalmus* × *Pangasius bocourti*)	Brewer's yeast (*Saccharomyces cerevisiae*)	30, 45, 60 or 75%	9 months	45%	Improved growth performance & immune response No effect on feed efficiency, blood hematology & meat quality ↑ lysozyme activity & total immunoglobulin (Ig)	[225]

### 3.15 Distiller's grains or distiller's dried grains with solubles

Distiller's grains (DG) or distiller's dried grains with solubles (DDGS) are the byproducts of the distillation process of cereals for production of ethanol. These are alternate protein sources. Starch rich cereals including corn, cassava, rice, millet, wheat, barley and sweet potato are sources of DDGS. These are used potentially for ethanol production [147]. DDGS are rich in protein, fat and phosphorus with low cost and high yield. They contain 30–35% crude protein, 10% crude fat and about 11% crude fiber. It might be utilized as superior source of protein for production and breeding [148]. DDGS are nutritionally valuable due to B vitamins, an unknown growth promotor factor as well as saccharides obtained during the fermentation [149]. Excluding fiber, other ANFs are absent in DDGS which interrupt the health and growth of fishes, as present in other plant protein sources, e.g., SBM, CSM, CM or SFM [150,151]. Further processing increases protein content in DDGS producing higher protein (∼40%) distillers dried grains (HP-DDGS). Annually over 40 million MT DDGS are obtained from the process of distillation in the United States alone and are a promising source for aquafeeds [152]. DDGSs have yeast as well that is a potential source of nucleotides and betaglucans, that may boost immune system in fish. Attempts have been done to utilize DDGS in aquaculture sector and its propriety has been checked in both in salmonids i.e. *O. mykiss* and omnivorous species i.e. tilapia with positive effects [153,154] (Table 15). However, more research studies are needed to find the optimum proportions of DDGS for aquafeed.

**Table 15:** Distiller's grains as substitute for fishmeal in diet of different fish species.

Fish species	Distiller's grains	Tested Inclusion levels	Duration	Optimum inclusion Level	Effects	Reference
Hybrid grouper *(E. fuscoguttatus ♀ × E. lanceolatus ♂)*	Corn distillers dried grains with solubles (DDGS)	6.67, 13.33, 20, 26.67 & 33.33%	56 days	7.80%	↑WGR & SGR upto 13.33% ↓ Amylase activity in gut, ↓mRNA levels of anti-inflammatory factors *tgf-β1 & il-10*, ↑mRNA levels of pro-inflammatory factors il-1β & tnf-α, microvilli damage = ↑DDGS	[226]
Grass Carp (*Ctenopharyngodon idellus*)	Dried Distillers Grains with Solubles (DDGS)	Native DDGS20 & DDGS30, US-imported DDGS20 & DDGS30	60 days	US-imported DDGS30	US-imported DDGS30 = ↑ growth via regulating genes involved in myogenesis & hypertrophy ↑Raw muscle collagen but negatively influence antioxidant capacity & cooked muscle texture	[227]
Yellow Perch (*Perca flavescens)*	High-protein distillers dried grains (HP-DDG)	25, 50, 75 & 100%	105 days	50%	↓Crude protein content & amino acids = ↑HP-DDG = ↓ growth	[228]
Common Carp (*C. carpio*)	Distillers dried grains with solubles (DDGS)	0, 25, 35%	49 days	_	No effects on growth parameters, flesh quality & blood biochemical profile, ↓ in liver tissue, CAT, GSH, MDA & carbonylated protein Variation in microbial density	[229]
Striped catfish (*P. hypophthalmus*)	High protein distillers dried grains	0, 25, 50 & 75%	98 days	25%	↓SGR, ↑FCR, ↓whole-body protein content & plasma proteins, ↑lipids	[230]
Turbot (*S. maximus)*	Corn distillers dried grains with solubles	10, 17.5, or 25%	84 days	_	↓Growth performance & impaired overall nitrogen & energy metabolism	[231]
Minor carp (*Labeo bata)*	Distiller's grain soluble	Brewery waste (test diet) &SBM (reference diet)	60 days	_	No effect on survival, growth parameters & biochemical composition of whole body tissue by brewery waste ↓ Feed cost	[232]

WGR = weight gain rate, SGR = specific growth rate, CAT = catalase, GSH = glutathione, MDA = malondialdehyde, SBM = soybean meal.

## 4 Conclusion

Natural conventional as well as non-conventional plant products are receiving more attention as promising substitutes due to their cheap availability in large quantities and predictable supply all year round but they are mostly replaced partially at specific levels in aquafeed. Above their optimum level of replacement, they exert negative effects on fish growth performance, body composition, metabolic activities and other biological parameters due to their ANFs. These factors may reduce by different methods such as heating, washing, solvent extraction, fermentation and enzymatic pretreatments. Also, incorporation of enzymes or bacteria into the plant proteins may increase feed utilization as these bacteria and enzymes stabilize the ANFs in the intestine of fish. Plant use in fish feed also establishes a good relationship between aquaculture and agriculture and can provide augmented production of aquaculture in a cost effective, eco-friendly and sustainable way. In the future, competition will increase for natural resources due to climate change, population growth and bio-economy development. In this case, aquaculture will play a key role in fulfilling the global demands for protein, elaborating innovating technology and exploring the use of alternative sustainable feed ingredients.

## 5 Future recommendations

**Fig. 14:**
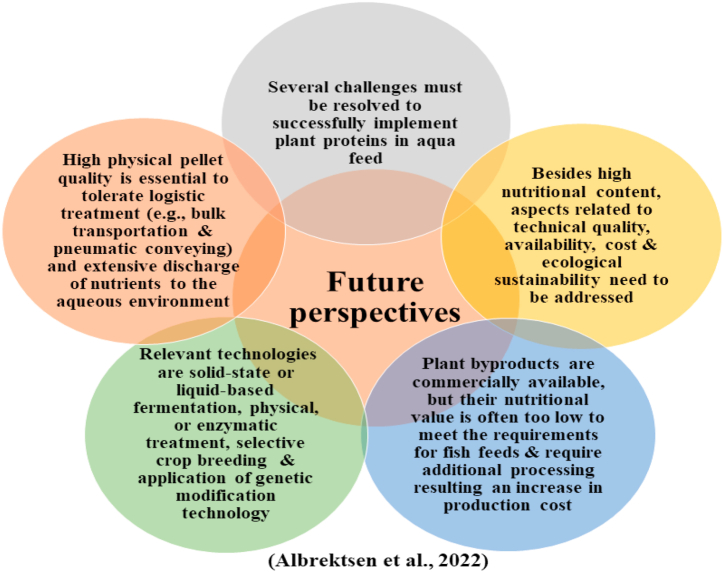
Future perspectives of using plant-based protein sources.

There is an urgent need to find compatible and efficient techniques by which these negative factors are removed from plant products and improving their digestibility and palatability as FM. Moreover, alternative plant protein sources and specific methods and techniques to optimize the nutritional composition of plant feeds should be determined. Additionally, research should also look at potential nutritional issues in fishes caused by consuming more plant proteins. Furthermore, by collating all the available knowledge, establishing a coordinated research effort through strategic planning is necessary to improve the nutritional content of plant feed ingredients in anticipation of increased usage in aquaculture. While plant components might lower feed prices, relevant economic strategy and incentive are needed to encourage feed millers to produce feed at lower costs. These measures will ultimately minimize the cost and dependence on naturally-sourced aquatic feed while increasing sustainability and resilience in aquacultural production (Fig. 14).

## Authors declaration statement

According to all authors, there is no conflict of interest with the publication of this paper.

## Role of funding source

The authors are grateful to the 10.13039/501100004681HEC Pakistan for funding Projects No. 20–4892/NRPU/R&D/HEC/14/1145 and 5649/Punjab/NRPU/R&D/HEC/2016 at the Department of Zoology, 10.13039/501100017152Government College University Faisalabad, Pakistan, for the provision of facilities. We thank the 10.13039/100008798Cynthia and George Mitchell Foundation, University of California, Santa Cruz, United States, for partially supporting the work. We also thank the University of California Santa Cruz, Dean of Social Sciences and Executive Vice Chancellor.

## Ethical statement

No ethical approval is required for this research.

## Data availability statement

Not applicable.

## CRediT authorship contribution statement

**Syed Makhdoom Hussain:** Writing – original draft, Supervision, Investigation, Conceptualization. **Aumme Adeeba Bano:** Writing – original draft. **Shafaqat Ali:** Writing – review & editing, Software, Data curation. **Muhammad Rizwan:** Writing – review & editing, Software, Data curation. **Muhammad Adrees:** Writing – review & editing, Software, Data curation. **Ameer Fawad Zahoor:** Writing – review & editing, Software, Data curation. **Pallab K. Sarker:** Writing – review & editing. **Majid Hussain:** Writing – review & editing. **Muhammad Zubair-ul-Hassan Arsalan:** Writing – review & editing. **Jean Wan Hong Yong:** Funding acquisition. **Adan Naeem:** Writing – review & editing.

## Declaration of competing interest

The authors declare that they have no known competing financial interests or personal relationships that could have appeared to influence the work reported in this paper.
